# Maternal predictors and quality of umbilical cord blood units

**DOI:** 10.1007/s10561-017-9657-y

**Published:** 2017-08-19

**Authors:** Beata Bielec-Berek, Żaneta Jastrzębska-Stojko, Agnieszka Drosdzol-Cop, Cecylia Jendyk, Dariusz Boruczkowski, Tomasz Ołdak, Agnieszka Nowak-Brzezińska, Rafał Stojko

**Affiliations:** 1 School of Health Sciences in Katowice, Medical University of Silesia Katowice, ul. Medyków 12, 40-752 Katowice, Poland; 2 Department of Anaesthesiology and Intensive Therapy, UCK prof. K. Gibińskiego, Medical University of Silesia, Katowice, ul. Ceglana 35, 40-514 Katowice, Poland; 3 Department of Obstetrics and Gynecology, The Boni Fratres Catoviensis Hospital, ul. Ks. Leopolda Markiefki 87, 40-211 Katowice, Poland; 4 Polish Stem Cells Bank, Warsaw, Poland; 5 Institute of Computer Science, Faculty of Computer Science and Material Science, Silesian University, ul. Bedzinska 39, 41-200 Sosnowiec, Poland

**Keywords:** Steam cell, Umbilical cord blood, Maternal age, Cord blood banking, Vaginal birth, Caesarean section

## Abstract

The aim of the study was to determine the relationship between the maternal age at delivery and selected properties of the cord blood stem cells. The study included 50 pregnant women aged between 18 and 38 years in which spontaneous labors or elective cesarean sections were performed. Umbilical cord blood was collected immediately after the women were delivered of newborns. The samples were analyzed in the Polish Stem Cells Bank in Warsaw. The highest mean WBC level (*p* < 0.05) was observed in the umbilical blood collected from patients aged 35 years and more. Similarly, the highest mean cell viability was observed in the umbilical cord blood collected from patients aged 35 and more. There were no statistically significant correlations between the CD34+ cells count and mean cell viability in the umbilical cord blood and the maternal age. With the significance level at *p* < 0.001, the females after spontaneous labor revealed a visibly higher WBC level than patients after a cesarean section. The higher mean WBC concentration (24.95 thousand/μl) was observed in the umbilical cord blood of patients aged 35 and more after spontaneous labors. In the same group, the umbilical cord blood was also characterized by the highest mean cell viability (98.72%). The number of nucleated cells in the umbilical cord blood collected in the perinatal period increases together with the maternal age. In the course of physiological spontaneous labors, the collected umbilical cord blood has more nucleated cells as compared with elective caesarian sections.

## Introduction

Umbilical cord blood stem cells are characterized by their high proliferation potential as well as the fact that their genome does not reveal any signs of aging and the risk of DNA mutation accumulation is significantly lower than in mature cells. Due to their increased telomerase activity, they reveal a faster rate of division and lower maturity (Pojda et al. 2003). Furthermore, the umbilical blood contains more immature, naïve T lymphocytes and therefore the umbilical cord blood stem cells transplantation is burdened with a lower risk of Graft-Versus-Host Disease (GVHD). Due to the lower requirements regarding the HLA compatibility, it is possible to perform the umbilical cord blood (UCB) transplantation from a non-related donor, which increases the chance of finding a donor (Apperley et al. 2008; Szabolc et al. 2003). Umbilical cord blood stem cells may in theory be obtained in the course of every single delivery. The procedure is fast, non-invasive and safe both for the newborn and the mother, and the material processing and storage are relatively simple. The blood frozen in liquid nitrogen is biologically stable (Stojko and Witek 2005). What matters, the application of umbilical cord blood stem cells does not rise controversies on ethical grounds. UCB constitutes a waste material after childbirth (Bradley and Cairo 2005).

Prior to freezing, the blood is tested in terms of nucleated cells, CD34+, their viability and the virological purity. Furthermore, each sample undergoes typing in terms of the HLA system (Butler and Menitove 2011; Machaj et al. 2001; Ołdak et al. 2000). For patients requiring therapy with stem cells, public UCB banks constitute a significant source of transplantation material. Therefore, public banks aim at collecting as much UCB as possible. Wide-scale research studies have been conducted which aimed to develop optimal conditions for obtaining umbilical cord blood, which in turn, ensures the highest viability of stem cells.

The primary aim of the conducted study was to determine the correlation between the maternal age and selected properties of umbilical cord blood stem cells. The study aimed to find the optimal maternal age for the collection of the highest “quality” material. Furthermore, the correlation between the mode of delivery, maternal age and transplantation quality of obtained material was evaluated.

## Materials and methods

The study covered 50 pregnant females aged between 18 and 38 years in whom either a spontaneous labor or an elective caesarean section were performed. The patients were divided into four age groups: women in labor between 18 and 25 years old; women in labor between 26 and 30 years old; women in labor between 31 and 35 years old; women in labor at the age of more than 35 years.

The study covered female patients who expressed informed consent to participate according to the protocol of the Bioethical Committee of the Medical University of Silesia in Katowice, Poland. Moreover, the accepted women presented a normal course of pregnancy, had not suffered from any other non-gynecological chronic disorders and had a spontaneous delivery or an elective caesarian section. In the studied group, there were 33 spontaneous labors (67% of all women), while in 17 women the elective caesarian section had been performed before spontaneous uterine contractions appeared.

Umbilical cord blood was collected directly after delivery, clamping and cutting of the umbilical cord of the newborn. The material was collected by puncturing the cord vessels and then it was placed into a plastic bag found in the collection kit containing 29 mg of anticoagulant CPD (Citrate Phosphate Dextrose Solution). Next, the blood was placed in special stabilizing gels and transported within 24 h at room temperature to the Polish Stem Cells Bank (PBKM) laboratory in Warsaw. At the laboratory, the material underwent a preparatory procedure in the course of which the cord blood unit volume was reduced via elimination of part of the autologous plasma and red blood cells. To eliminate red blood cells, their sedimentation was performed in an HES 6% solution (Grifols), and to eliminate the autologous plasma, the material was centrifuged for 15 min at 20 °C at 1420×*g*.

For the purpose of the study, approximately 0.5 ml was collected from the obtained suspension of nucleated cells and the transplantation quality of umbilical cord blood was determined.

The count of nucleated cells was performed with the automated hematology analyzer MICROS 60 (Horiba ABX). Quantification was performed twice, prior to the material preparation and after completing it, which enabled to determine the proportional cell recovery.

The enumeration of the CD34+ hematopoietic stem cells was performed with the flow cytometry unit FACS Calibur. The cell suspension was labelled with phycoerythrin (PE)-conjugated monoclonal antibodies which were directed against HPCA-2 antigens (Becton–Dickinson) and fluorescein (FITC)-conjugated monoclonal antibodies against CD45 antigens. The control group consisted of cells labelled with polyclonal antibodies. The total hematopoietic cells count was determined on the basis of the proportion of cells determined with the flow cytometry and the nucleated cells count was obtained with the hematology analyzer.

In order to evaluate their viability, the umbilical cord blood stem cells were labelled with 7-amino-actinomycin D (7-AAD) (Via Probe, BD). 7-AAD is a compound with a strong affinity for DNA. This agent passes only through dead cells and stains them. The cells were analyzed with the FACS Calibur flow cytometer and the CellQUEST Pro, BD software. Viability was assessed on the basis of the proportion of cells not stained with 7-AAD.

## Statistical analysis

The obtained results were collected in an MS Office Excel spreadsheet and then translated into Statistica PL software. Next, the ANOVA variance analysis was performed. Statistical significance was assumed at *p* < 0.05.

## Results

The mean cell viability of the collected umbilical cord blood units is 97.15 ± 2.30%. The highest cell viability is 99.88%, while the lowest recorded in the studied material was 86.71%.

In 48% of the studied females, the cord blood cells are characterized by viability higher than 98%, while in only 12% of cases the viability is lower than 95%.

While analyzing the viability of CD34+ cells in the studied material it was observed that in the majority of cases (82% of the obtained cord blood) the proportion of hematopoietic cells is between 0.1 and 0.5, while the mean value of the CD34+ cells in the umbilical cord blood obtained after the birth was 0.24 ± 0.15.

On the other hand, the concentration of nucleated cells in the studied material ranges between 7.80 and 27.20 thousand/μl. The vast majority (80%) of the collected cord blood reveals the level of WBCs above 10 thousand/μl.

The mean maternal age was 28.86 years (SD = 4.54). The most numerous (40%) was the group of females aged between 26 and 30. Women aged 35 or more constituted the smallest group (12% of the studied population).

The effect of the maternal age on the following was evaluated: the number of nucleated cells, nucleated cells viability and the concentration of CD34+ hematopoietic stem cells in umbilical cord blood.

The results revealed that the number of WBCs in the umbilical cord blood statistically significantly depends on the maternal age, which is shown in Table 1. The highest mean WBC level (*p* < 0.05) was observed in the umbilical blood collected in the course of delivery from patients aged 35 years and more. On the other hand, the lowest mean WBC concentration was found in the umbilical cord blood drawn from patients aged between 18 and 25.

**Table 1:** Assessment of the correlation between cell viability, WBC and CD34+ cells counts in umbilical cord blood and maternal age

Age (years)	CD34+ (amount/μl)	%CD34+	Cell viability (%)	WBC (thousand/μl)
	Mean ± SD Min–Max	Mean ± SD Min–Max	Mean ± SD Min–Max	Mean ± SD Min–Max
18–25 years old	106.58 ± 83.57 10.0–316.0		96.46 ± 3.76 86.71–99.88	10.96 ± 2.46 8.00–16.6
26–30 years old	147.0 ± 83.20 49.0–352.0		97.55 ± 1.62 94.04–99.87	14.29 ± 3.47 8.3–23.7
31–35 years old	95.5 ± 54.08 17.0–245.0	0.19 ± 0.11 0.03–0.49	96.81 ± 1.75 92.88–98.66	14.65 ± 3.78 9.0–21.8
35 years old and more	103.83 ± 20.41 68.0–121.0	0.21 ± 0.04 0.14–0.24	97.86 ± 1.17 96.28–99.65	16.47 ± 7.57 7.8–27.2
Total	119.76 ± 73.97 10.0–352.0	0.24 ± 0.15 0.03–0.70	97.15 ± 2.30 7.16–7.48	13.84 ± 4.27 7.8–27.2
*p*	Ns (*p* = 0.199470)	Ns (*p* = 0.173606)	Ns (*p* = 0.482674)	0.032913

Similarly, the highest mean cell viability was observed in the umbilical cord blood collected from patients aged 35 and more. UCB cells viability in this age group was never lower than 96.28%, with its peak at 99.65%. The lowest mean cell viability was reported in women aged between 18 and 25 and it equaled 96.46%. Nevertheless, these differences were not statistically significant.

Additionally, there was no statistically significant correlation between the CD34+ cells count in the umbilical cord blood and the maternal age.

In the studied group, 33 patients delivered in the spontaneous manner (which constitutes 67% of all patients), while in 17 women the elective caesarian section was performed. The patients, depending on age and the mode of delivery, did differ statistically significantly in terms of WBC concentration in umbilical cord (Table 2).

**Table 2:** Assessment of the correlation between cell viability, CD34+ cells and WBC concentration in umbilical cord blood and the mode of labor and the maternal age

Mode of delivery	Age (years)	CD34+ (amount/μl)	%CD34+	WBC (thousand/μl)	Cell viability (%)
		Mean ± SD (Min–Max)	Mean ± SD (Min–Max)	Mean ± SD (Min–Max)	Mean ± SD (Min–Max)
Spontaneous labor	18–25 years old	104.75 ± 91.25 34.0–316.0	0.21 ± 0.18 0.07–0.63	11.04 ± 2.64 8.60–16.60	95.52 ± 4.10 86.71–99.23
	26–30 years old	147.41 ± 84.13 49.0–352.0	0.30 ± 0.17 0.10–0.70	14.77 ± 3.41 10.40–23.70	97.42 ± 1.72 94.04–99.87
	31–35 years old	84.43 ± 19.20 52.0–106.0	0.17 ± 0.04 0.10–0.21	15.44 ± 2.51 12.00–19.10	96.96 ± 2.13 92.88–98.66
	35 years old and more	94.5 ± 37.48 68.0–121.0	0.19 ± 0.07 0.14–0.24	24.95 ± 3.18 22.70 ± 27.20	98.72 ± 1.32 97.78–99.65
C-section	18–25 years old	110.25 ± 78.44 10.0–188.0	0.26 ± 0.11 0.16–0.38	10.80 ± 2.43 8.00–13.90	98.36 ± 2.33 94.88–99.88
	26–30 years old	144.67 ± 95.52 69.0–252	0.29 ± 0.19 0.14–0.50	11.53 ± 2.82 8.30–13.50	98.27 ± 0.66 97.75–99.01
	31–35 years old	111.0–84.52 17.0–245.0	0.22 ± 0.17 0.03–0.49	13.54 ± 5.21 9.00–21.80	96.59 ± 1.23 95.03–98.29
	35 years old and more	108.5 ± 11.79 97.00–121.0	0.22 ± 0.02 0.19–0.24	12.23 ± 4.50 7.80–18.00	97.44 ± 0.98 96.28–98.35
Total groups		119.76 ± 73.97 10.0–352.0	0.24 ± 0.15 0.03–0.70	13.84 ± 4.27 7.80–27.20	97.15 ± 2.30 86.71–99.88
*p*		Ns (*p* = 0.673644)	Ns (*p* = 0.601288)	0.000340	Ns (*p* = 0.398127)

With the significance level at *p* < 0.001, the females after spontaneous labor revealed a visibly higher WBC level than patients after a C-section. The higher mean WBC concentration (24.95 thousand/μl) was observed in the umbilical cord blood units of patients aged 35 and more after spontaneous labors. In the same group, the umbilical cord blood was also characterized by the highest mean cell viability (98.72%). Viability of blood cells collected from these patients was never lower than 97.78%. However, these differences were not statistically significant.

In patients after elective C-sections, the highest WBC concentration in umbilical cord blood was observed among women aged between 32 and 35 years.

The mode of labor and the maternal age did not have any statistically significant effect on the concentration of CD34+ cells in the umbilical cord blood. However, it may be noted that both in the group of the women after spontaneous labors and elective caesarian sections the highest concentration of CD34+ cells in the umbilical cord blood was observed among those aged between 26 and 30 years.

## Discussion

Biological properties of stem cells as well as their potential clinical application place them in the center of attention in the scientific world. Bone marrow and peripheral blood are the best known sources of stem cells. However, the umbilical cord blood—as an alternative source of stem cells—has fascinated scientists for many years. There are centers all over the world which specialize in storing umbilical cord blood stem cells collected in the perinatal period which may be used for auto- and allo-transplantation purposes. For patients who require therapy with stem cells public banks of umbilical cord blood constitute a significant source of tested and ready-to-use transplantation material. It is necessary to undertake attempts aimed at developing optimal conditions of collecting umbilical cord blood so that the stored material is of the highest transplantation quality.

An interesting issue that has not been frequently investigated so far is the relationship between the maternal age and the viability and the number of stem cells. Our own studies reveal that there is a statistically significant correlation between the maternal age and the nucleated cells count in the umbilical cord blood. The highest mean WBC concentration was observed in the umbilical cord blood collected after the birth from women aged 35 and more. On the other hand, no statistically significant differences were found between the maternal age and the cell viability and the number of CD34+ cells. McGuckin et al. (2007) showed a statistically significant dependence between the maternal age and the concentration of hematopoietic cells in the umbilical cord blood. The older the women from whom umbilical cord blood was collected, the lower the mean concentration of HSC cells in the material. However, while investigating the mean level of nucleated cells it was observed that women who give birth before the 20th and after the 37th year of age are characterized by a lower mean number of WBCs in the umbilical cord blood collected in the course of labor as compared with women within this age range. Nakagawa et al. (2004) observed that higher percentage of CD34+ cells in the umbilical cord blood is related to the younger maternal age. On the other hand, Mohyeddin et al. (2004) observed that the umbilical cord blood collected in the course of labor from women older than 25 years of age contains statistically significantly more nucleated cells. However, the Ballen’s team did not observe the maternal age to have any effect on the laboratory parameters of umbilical cord blood collected in the perinatal period. Furthermore, children born by mothers aged between 35 and 40 years of age did not lose the hematopoietic potential (Ballen et al. 2001).

Another studied aspect was the effect of the mode of labor (spontaneous vs. elective caesarian section) on selected parameters of umbilical cord blood. The increased energy demand and the increased metabolic activity which result from the intensified activity of uterus muscles and skeletal muscles as well as accompanying oxygen consumption lead to increased production of reactive forms of oxygen (intensified oxidative stress) in the course of labor. In this scenario, the organism responds with an increase of stem cells in the blood of the fetus, i.e. also in the umbilical cord blood. These assumptions are confirmed by Aufderhaar et al. (2003) who showed that the perinatal factors, such as low blood pH and prolonged 1st stage of labor correlate with the increase in the number of nucleated cells, granulocytes, CD34+ cells and progenitor cells in umbilical cord blood. Similarly, Shelbak et al. (1998) confirms that low pH positively correlates with the number of MNCs, and the number of CFU-GM increases proportionally to the length of the first stage of labor.

It has been suggested that spontaneous labors are accompanied with a more intensified oxidative stress. A statistically significantly higher WBC concentration in the umbilical cord blood collected in the course of spontaneous labors was observed as compared with elective caesarian sections (Mohyeddin et al. 2004; Mancinelli et al. 2006; Omori et al. 2012; Sparrow et al. 2002; Nikischin et al. 1997). Additionally, a higher number of hematopoietic cells was observed in the umbilical cord blood after spontaneous labors (Sparrow et al. 2002). However, Molloy et al. (2004) observed that after the spontaneous labor there is an increased leukocyte resistance to apoptosis, while white blood cells obtained after caesarian sections did not react to polysaccharides, which suggests their activity is reduced. The most convincing evidence can be found in research studies which assess the activity of antioxidative enzymes, oxidative stress markers or the level of hormones or cytokines. A significantly higher concentration of malondialdehyde (MDA) and WBCs accompanies spontaneous labors. On the other hand, a higher activity of superoxide dysmutase and catalase was observed in blood collected in the course of C-sections (Inanca et al. 2005). Mears et al. (2004) observed an increased concentration of cortisol in the umbilical cord blood obtained in the course of spontaneous labor as compared with material collected while performing C-sections. Similarly, the umbilical cord blood from spontaneous labors was characterized by higher concentrations of adrenalin, noradrenalin and ACTH (Vogl et al. 2006). On the other hand, the team of Malamitsi-Puchner et al. (2005), while comparing the level of cytokines depending on the mode of labor, observed a significantly higher level of pro-inflammatory cytokines (IL-1, TNFα, INFγ) in the umbilical cord blood after spontaneous labors. The above-discussed papers seem to confirm our own results, which reveal a statistically significant difference in the amount of WBCs in umbilical cord blood depending on the mode of labor. A much higher mean concentration of nucleated cells can be found in the tissue material obtained in the course of spontaneous labors. The highest mean WBC concentration was reported in the umbilical cord blood collected in the perinatal period from women after spontaneous labors aged 35 years and more. No statistically significant differences in the cell viability or the CD34+ cells count were observed.

However, there is a group of scientists who observe in their studies a higher concentration of nucleated cells in the umbilical cord blood collected in the course of C-sections (Nunes and Zandavalli 2015). Szymańska-Toczek et al. (2001) reported a higher concentration and activity of neutrophils after C-sections.

In the past, the umbilical cord blood together with the placenta were a medical waste which was discarded after each delivery. Nowadays, it is well known that this material conceals a huge transplantation potential in the form of stem cells. The ease of collecting, its safety for both the mother and the child, a low risk of GvHD and a low risk of transmitting an infection constitute additional advantages of using the umbilical cord blood for transplantation purposes. Therefore, the umbilical cord blood constitutes an alternative—and in many countries the main—source of stem cells in the treatment of diseases of the hematopoietic and immunological systems and some genetic disorders. The only limitation for this type of transplantation is the concentration of stem cells in the obtained tissue material. At present, numerous variables which may have an effect on the “quality” of umbilical cord blood constitute the subject of multiple studies. Gaining insight into these factors will allow to develop more precise conditions of collecting, processing and storing the umbilical cord blood so that the obtained material is characterized by the highest “quality”, and also will reduce the financial outlay to the necessary minimum. Moreover, studies on controlling the process of stem cell differentiation or the mechanisms controlling the stem cells differentiation at the molecular level will provide data which will be used in the future in the treatment of diseases which remain incurable so far. All these activities are aimed at increasing the post-transplantation success—engraftment and full recovery of the patient. Hopes and prospects for the use of stem cells in the future are immense.

## Conclusions

The number of nucleated cells in the umbilical cord blood collected in the perinatal period increases together with the maternal age. In the course of physiological spontaneous labors, the collected umbilical cord blood has more nucleated cells as compared with elective caesarian sections.
